# Confocal Laser Scanner Evaluation of Bactericidal Effect of Chitosan Nanodroplets Loaded with Benzalkonium Chloride

**DOI:** 10.3390/jcm11061650

**Published:** 2022-03-16

**Authors:** Mario Alovisi, Damiano Pasqualini, Narcisa Mandras, Janira Roana, Pietro Costamagna, Allegra Comba, Roberta Cavalli, Anna Luganini, Alfredo Iandolo, Lorenza Cavallo, Nicola Scotti, Elio Berutti

**Affiliations:** 1 Department of Surgical Sciences, Dental School, University of Turin, 10126 Turin, Italy; damiano.pasqualini@unito.it (D.P.); costamagna@hotmail.it (P.C.); allegra.comba@unito.it (A.C.); nicola.scotti@unito.it (N.S.); elio.berutti@unito.it (E.B.); 2 Department of Public Health and Pediatrics, University of Turin, 10126 Turin, Italy; narcisa.mandras@unito.it (N.M.); janira.roana@unito.it (J.R.); lorenza.cavallo@unito.it (L.C.); 3 Department of Drug Science and Technology, University of Turin, 10126 Turin, Italy; roberta.cavalli@unito.it; 4 Department of Life Sciences and Systems Biology, University of Turin, 10126 Turin, Italy; anna.luganini@unito.it; 5 Department of Medicine, Surgery and Dentistry, Salerno Medical School, University of Salerno, 84084 Salerno, Italy; iandoloalfredo@unisa.it

**Keywords:** nanodroplets, confocal laser microscope, benzalkonium chloride, chlorhexidine, sodium hypochlorite, viability staining

## Abstract

The aim was to evaluate the antibacterial efficacy and penetration depth into dentinal tubules of a solution of chitosan nanodroplets (NDs) loaded with Benzalkonium Chloride (BAK). Seventy-two human single-root teeth with fully formed apex were used. Cylindrical root dentin blocks were longitudinally sectioned and enlarged to a size of a Gates Glidden drill #4. After sterilization, root canals were infected with *Enterococcus faecalis* ATCC 29212 and further incubated for three weeks. Specimens were assigned to three experimental groups (*n* = 20), plus positive (*n* = 6) and negative (*n* = 6) controls. In the first group, irrigation was achieved with 2 mL of NDs solution loaded with BAK (NDs-BAK), in the second with 2 mL of 5% sodium hypochlorite (NaOCl) and in the last with 2 mL of 2% chlorhexidine (CHX). Specimens were rinsed and vertically fractured. Confocal laser scanning microscopy (CLSM) and viability staining were used to analyze the proportions of dead and live bacteria quantitatively. The volume ratio of red fluorescence (dead) was calculated in 3D reconstructions. Data were analyzed by one-way ANOVA and post hoc Bonferroni tests (*p* < 0.05). The ratio of red fluorescence over the whole green/red fluorescence resulted in a significant comparison of NDs-BAK with NaOCl (*p* < 0.01) and NaOCl with CHX (*p* < 0.01). No differences were found between NDs-BAK and CHX (*p* > 0.05). The mean depth of efficacy was, respectively: NDs-BAK 325.25 μm, NaOCl 273.36 μm and CHX 246.78 μm with no statistical differences between groups. The NaOCl solution showed the highest antimicrobial efficacy, but nanodroplets with BAK seemed to have the same effect as CHX with a high depth of efficacy.

## 1. Introduction

The prevalence of apical periodontitis among adult populations is more than 35–40% and increases with age [1,2]. Although epidemiological studies reported an elevated endodontic treatment success rate, many apical periodontal lesions seem to affect previously root-filled teeth [2,3].

To achieve long-term success, removing the pulp tissue remnants, bacteria and microbial toxins from the root canal system is essential. However, nickel--titanium (NiTi) rotary instrumentation only acts on the central root canal volume, leaving lateral canals and isthmuses untouched after preparation [4,5]. Therefore, microorganisms remaining in the root canal system after treatment or re-colonizing the filled canal system are considered an important cause of endodontic failure [6].

Recent microcomputed tomography (micro-CT) studies have demonstrated that large areas of the root canal walls remain untouched, emphasizing the importance of chemical irrigation [5,7,8]. Nevertheless, irrigation is an essential part of root canal debridement because it allows for cleaning beyond what might be achieved by root canal instrumentation alone [9,10].

The remarkable reduction in preparation size and taper and treatment time for even simpler and minimally invasive shaping protocols encourages the development of more efficient irrigation systems [11]. Sodium hypochlorite (NaOCl) is the most used irrigant during root canal preparation due to its high capacity for tissue dissolution and a well-known antimicrobial activity [12]. Surfactants are associated with NaOCl to improve its properties, such as dissolution of organic tissues, antimicrobial activity and penetration into the root canal system [13].

Many other antimicrobial agents have been proposed for root canal irrigation, such as chlorhexidine (CHX) and benzalkonium chloride (BAK) [14,15]. The latter is a cationic detergent composed of quaternary ammonium. BAK causes structural disorganization, loss of cytoplasmic membrane integrity and other destructive effects against bacteria [16]. Moreover, in concentrations up to 5%, it is claimed to provide antibacterial properties and long-lasting effects due to its ability to inhibit proteases [17].

The effectiveness of BAK administration deep into the root canal and tissues, coronal through the dentinal microtubules, could be augmented using nanodroplets (NDs) [18]. Experimental NDs with chitosan shells have been recently proposed [19]. Chitosan (CS) is a nontoxic biopolymer derived by the deacetylation of chitin and, due to its catatonic nature, it has broad antimicrobial activity against bacteria, with a high killing rate through interaction with the bacterial cell wall [20]. This interaction between chitosan and the bacterial cell depends on the hydrophilicity of the cell wall, which could explain the lower toxicity of chitosan to mammalian cells [18,19,20]. Therefore, NDs with chitosan shells offer different advantages such as a broad spectrum of antibacterial activity, biocompatibility and the ability of a long-lasting release of antimicrobial substances [21,22]. However, no studies are available about the potential in vitro efficacy of a solution of NDs loaded with BAK for the improvement of the endodontic disinfection.

Recent studies have visualized bacteria in dentinal tubules by confocal laser scanning microscopy (CLSM), which has been reported to be an appropriate way, not just to visualize bacteria, but also to identify live and dead bacteria in the infected dentin [23,24]. 

This study evaluated the antibacterial efficacy and depth of penetration into dentinal tubules of a solution of NDs loaded with BAK through CLSM images compared with NaOCl and CHX. 

## 2. Materials and Methods

### 2.1. Manufacturing of NDs and Characterization

A decafluoropentane nano-emulsion was obtained and stabilized with dipalmitoyl phosphatidylcholine and palmitic acid (1% *w*/*v*). Afterward, a chitosan solution (2.7% *w*/*v*) at pH = 5.5 was added dropwise under stirring. The NDs formulation was sterilized through ultraviolet (UV)-C ray exposure for 20 min. To evaluate sterilization efficacy, UV-C-treated NDs were incubated with cell culture Gibco Dulbecco’s Modified Eagle Medium (DMEM) in a humidified CO_2_/air incubator at 37 °C, up to 72 h. No microbial contamination was observed when NDs and FITC-labeled NDs were checked by optical microscopy. Physic-chemical characterization of chitosan-shelled/decafluoropentane-cored NDs formulation with BAK was performed in vitro to evaluate the size, morphology and surface charge [19].

### 2.2. Antimicrobial Activity of Benzalkonium Chloride against E. faecalis

The antibacterial activity of NDs loaded with BAK was evaluated comparatively with the free BAK by quantitative analyses. *Enterococcus faecalis* ATCC 29212 was used as the model organism. The broth microdilution assay for quantitative analysis was carried out in accordance with the CLSI M100-S27 to determine the lowest concentration (minimal inhibitory concentration, MIC) and the minimum bactericidal concentration (MBC) of NDs loaded with BAK and free BAK, which inhibit the growth of bacteria.

Seventy-two human single-root teeth with fully formed apex were extracted for periodontal reasons and stored in 4% thymol solution. All samples were collected with informed consent and the Ethical Committee of the University of Turin approved the study protocol (Approval code: DS_00052_2021; Approval date: 20 June 2021). A root dentin block of 4 mm was horizontally sectioned from 1 mm below the cementoenamel junction by a 0.5 mm-thick diamond saw (Isomet 5000; Buehler Ltd., Lake Bluff, IL, USA) at 1000 rpm under water cooling. A Gates Glidden drill #4 (1.1 mm in diameter) (Tulsa Dentsply, Tulsa, OK, USA) at 300 rpm under water cooling was used to enlarge root canals. The smear layer was removed using EDTA 10% for 5 min. Specimens were then packaged and sterilized.

### 2.3. Irrigation Protocol

Sixty-six of 72 sterilized specimens were placed in multi-well support under a laminar flow biohazard cabinet (CLANLAF-VFR 1206, Racine, WI, USA). Six negative controls (C−) (without bacteria) were used to ensure the efficacy of the sterilization procedures. The root canals of 66 remaining specimens were infected, with an overnight culture of *E. faecalis* ATCC 29212, to match the turbidity of 3 × 108 colony-forming units/mL (CFU/mL) as confirmed by colony counts in triplicate, in Brain Heart Infusion (BHI; Oxoid, Milan, Italy) broth. They were further incubated aerobically with 5% CO_2_ at 37 °C for three weeks to allow penetration of *E. faecalis* into dentin tubules. The fresh broth was replaced every fourth day. The purity of the cultures was checked regularly. After three weeks of infection, a control group with six untreated samples was set up as positive control (C+), and the remaining specimens were randomly subdivided into three groups to compare the antimicrobial efficacy of nanodroplets charged with BAK to different types of disinfectants. In the group NDs-BAK (*n* = 20), irrigation was performed for 3 min with 2 mL of NDs solution loaded with BAK; in the group NaOCl (*n* = 20), irrigation was performed for 3 min with 2 mL of 5% NaOCl; and in the group CHX (*n* = 20), irrigation was performed for 3 min with 2 mL of 2% chlorhexidine (CHX).

Each cylindrical dentin block was fractured into two semi-cylindrical halves by making a thin groove in the middle of the specimen, using a low-speed handpiece with a small round bur (Tulsa Dentsply). The size of the fine specimen was about 4 × 4 × 2 mm, and the corresponding irrigant in each group was applied on the internal surface [25].

### 2.4. Confocal Laser Scanning Microscopy Analysis

Specimens’ root canals were accurately rinsed with sterile saline solution, and then Live/Dead Backlight (L/D) viability testing was used to detect viable and dead bacteria: Syto 9 (20/1) and PI (120/1) with a 1:1 ratio (20,000:20,000 µL). Specimens were immersed in the L/D viability test solution, agitated and stocked in a dark container for 30 min; the excess of L/D was then removed with repeated saline solution rinses. Specimens were stocked in a microscopy chamber after having positioned 100 µL of saline solution in the multi-well, and then stocked in a dark room until the CLSM examination. The specimens were then washed in sterile water for 1 min and vertically fractured through the root canal into two flat halves to expose a fresh surface of longitudinally visible dentin canals for CLSM examination [25]. CLSM analysis microscopy imaging was performed using a confocal Olympus IX70 (Olympus optical co. GMBH. Hamburg, Germany) Fluorescence Microscope with illumination by a Krypton/Argon laser (488 nm). Two detection channels captured Syto9 and PI. Emission wavelengths of 505–550 nm (green, Syto9) and 650–750 nm (red, PI) were collected to visualize Syto 9 and PI, respectively. Six additional uninfected samples were stained under the same protocol and used as negative controls to set channel thresholds and ensure standardization [25]. Images were taken using an HCX PL APO CS 20×/0.7 NA oil immersion objective with a different zoom of 2. The software ImageJ (NIH, Bethesda, MD, USA) acquired confocal laser scanning microscopic images at a 1024 × 1024 pixel scan area. For the tested samples, each group’s mean depth of action was calculated from ten separate measurements for every single image, adjusted for the red color channel. The mean ratio of red fluorescence over the whole red/green fluorescence (red fluorescence ratio), indicating the proportion of dead cells for each group, was calculated from merged images and three-dimensional reconstructions. This measurement was considered a surrogate marker of bactericidal efficacy.

### 2.5. Statistical Analyses

Mean depths of action were recorded, and differences were analyzed with one-way ANOVA and post hoc Bonferroni testing (*p* < 0.05). The Kolmogorov–Smirnov test for normality was used to analyze the data distribution of the antibacterial effect. The data were collected, and the differences among groups were analyzed by Kruskal–Wallis and Dunn’s post hoc test (*p* < 0.05).

## 3. Results 

The results for MIC and MBC for *E. faecalis* ATCC 29212 showed that BAK (0.023 µg/mL and 0.046 µg/mL, respectively), and NDs-BAK (0.046 µg/mL and 0.186 µg/mL, respectively), were strongly antibacterial. MIC and MBC of CHX were determined as 0.0037 µg/mL and 0.0015 µg/mL, respectively, which resulted in complete inhibition of bacterial growth.

**Figure 1:**
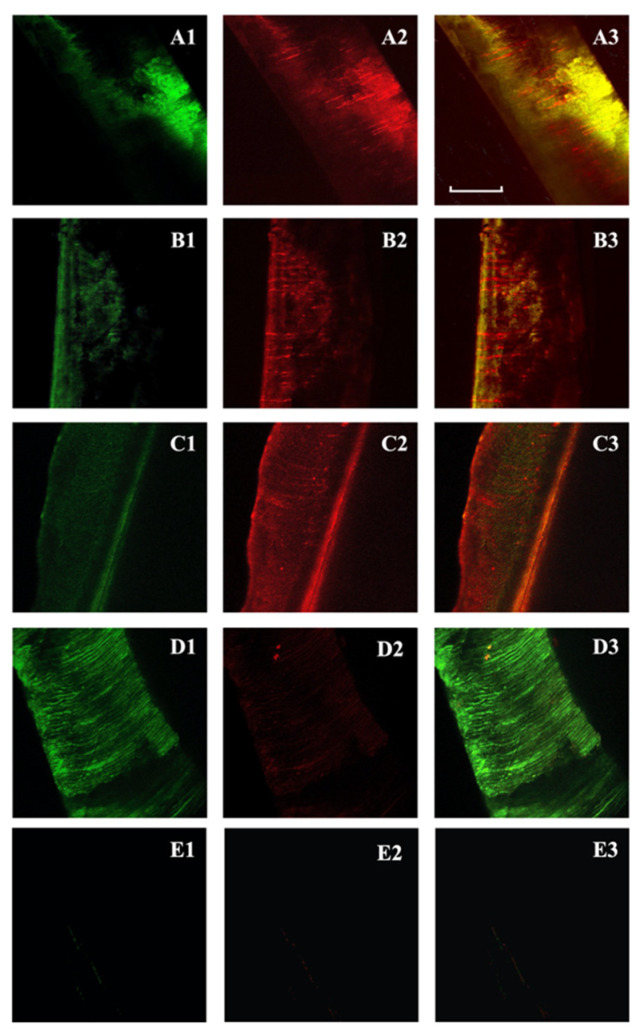
Confocal laser scanning microscopy of *Enterococcus faecalis*-infected dentinal tubules after different irrigation protocols and viability staining. Two-dimensional images of the green channel (**A1**–**D1**); two-dimensional images of the red channel (**A2**–**D2**); two-dimensional images of the composite reconstruction (**A3**–**D3**). NDs-BAK group (nanodroplets with Benzalkonium Chloride) (**A1**–**A3**). CHX group (chlorhexidine) (**B1**–**B3**). NaOCl group (sodium hypochlorite) (**C1**–**C3**). Positive controls (**D1**–**D3**) and negative controls (**E1**–**E3**). The pulpal side is represented on the left side for each image and the scale length is 300 μm.

The penetration of *E. faecalis* from the root canal side into the dentinal tubules was verified with CLSM. The negative control group showed no bacterial contamination. The mean depth of action and the mean proportion of dead cells volume (red fluorescence ratio) for each group are reported in Figure 1 and Table 1 and Table 2. The three groups exhibited similar mean depths of action: NDs-BAK 325.25 μm, NaOCl 273.36 μm and CHX 246.78 μm, respectively. It was slightly higher in NDs-BAK, but the differences were not statistically significant (*p* > 0.05).

The mean red fluorescence ratio(s) was higher in NaOCl (91.23%), whereas it was similar between the NDs-BAK and CHX groups (68.78% and 65.14%, respectively) (Table 1). The NaOCl solution showed higher antimicrobial efficacy, whereas nanodroplets with BAK seemed to have the same effect as CHX. There was a statistical difference comparing NDs-BAK with NaOCl (*p* < 0.01) and NaOCl with CHX (*p* < 0.001) related to the antimicrobial activity (Table 2). No differences were found between NDs-BAK and CHX (*p* > 0.05). The positive controls showed a lower mean fluorescence ratio and negative controls were also analyzed (Figure 1, Table 1).

## 4. Discussion

The experimental data reported that the innovative solution consisting of NDs loaded with BAK displayed a high depth of action inside dentinal tubules and an antibacterial efficacy comparable to CHX.

It has been shown that the success of endodontic treatment depends on two factors: the eradication of bacteria from the root canal system and the absence of reinfection [26,27]. During endodontic treatment, a central role is played by the irrigants that are used to clean the root canal and to eliminate the bacterial biofilm [28]. The most common way to eradicate this infection is to clean the root canal with a dilute solution of sodium hypochlorite [9,10]. Usually, its activity is time-dependent, and several agitation techniques have been proposed to activate irrigants and optimize root canal disinfection with modern low-tapered and time-sparing shaping techniques [9,10,11,29,30]. However, there is not a single irrigating solution that alone sufficiently covers all the functions required for an ideal root canal cleaning [9,10].

BAK is often referred to as a synthetic antimicrobial agent with a broad-spectrum antimicrobial function, and its activity could be increased by a delayed release deep inside dentinal tubules [15,16,18]. Therefore, this in vitro study evaluated the antibacterial efficacy and depth of action of an innovative solution of chitosan NDs loaded with BAK proposed for endodontic irrigation and with supposed long-lasting antimicrobial activity.

Previous studies reported a high antibacterial activity of BAK against Gram-positive and Gram-negative bacteria, the latter being slightly less sensitive [16,31]. *E. faecalis* is physically and ecologically strong, and it is often present in persistent endodontic infections. Therefore, it is widely used to test the effectiveness of the endodontic disinfecting agents [31]. Finally, Enterococcus’ shape is quite round, and they have a relatively small cell diameter, which makes it easier to force them into dentinal tubules [24,32].

After exposure to the antibacterial solutions, the infected dentin specimens were fractured to obtain a fresh dentin surface for CLSM analysis. The border of the fractured root dentin surface was first localized with the microscope to ensure a reproducible scanning field [25]. The bacterial presence could not be determined until the images were processed, ensuring a blindfold evaluation [25,33]. Moreover, representative data from all randomly selected areas with an excellent signal-to-noise fluorescence ratio were obtained due to the presence of bacteria in the dentinal tubules [25]. However, background fluorescence was occasionally observed within the canal lumen and the root canal samples showed auto-fluorescent materials not to be confused with bacteria [23,24,25]. Nevertheless, the background fluorescence intensity was minimal within the tubules and there was no inference with the signal generated from bacteria [33].

Confocal-laser scanning microscope visualized the presence of microorganisms in the root canal dentinal tubules due to its ability to penetrate below the surface of the specimen and to include the dentin canals that are not open on the surface [2,25,32,33].

Microorganism selection depends on the focus of the study and an endodontic biofilm consists mostly of Enterococcus faecalis. Usually, the common analyzed variables are counts of colony-forming units or the percentage of dead bacteria determined by confocal laser scanning microscopy after applying a differentiating stain. These models are helpful to evaluate new antimicrobial treatment options, even if a new therapy has yet to be proven in randomized controlled clinical trials [34].

In the present study, the CLSM analysis showed no statistical difference among groups concerning the depth of action, even if the NDs-BAK solution seemed to penetrate deeper on the limit of statistical significance. Previous studies showed lower BAK dentin tubular penetration, but the use of a NDs carrier could be beneficial for a deeper irrigant penetration [35,36]. The overall efficacy of the tested NDs-BAK solution was similar to CHX. These results seem in accordance with the available literature, despite the differences in methodology [17]. In conclusion, within the limitation of this in vitro study based on CLSM analysis, nanodroplets charged with BAK, although they failed to show the same antibacterial efficacy of NaOCl, proved as effective as the CHX solution, with deep penetration ability inside tubules [21,22].

## Figures and Tables

**Table 1:** Mean depth of action and antimicrobial activity (Red Fluorescence Ratio) of NDs-BAK (nanodroplets with Benzalkonium Chloride), CHX (chlorhexidine), NaOCl (sodium hypochlorite), C+ (positive controls) and C− (negative controls). Nd: data not determined.

	NDs-BAK	NaOCl	CHX	C+	C−
Mean Depth of Action (μm)	325.25 ± 134.52	273.36 ± 181.49	246.78 ± 75.88	0.52 ± 0	Nd
Red Fluorescence Ratio (%)	68.78 ± 0.0956	91.23 ± 0.1066	65.14 ± 0.1362	0.01 ± 0	Nd

**Table 2:** Comparison among groups of the tested parameters depth of action and antimicrobial effect. NDs-BAK (nanodroplets with Benzalkonium Chloride), CHX (chlorhexidine) and NaOCl (sodium hypochlorite). Level of statistical significance (*p* < 0.05).

	Mean Difference (Mean Depth of Action)	*p*-Value	Mean Difference (Red Fluorescence Ratio)	*p*-Value
NDs-BAK vs. NaOCl	11,493	*p* > 0.05	−20,131	*p* < 0.01
NDs-BAK vs. CHX	15,092	*p* > 0.05	2596	*p* > 0.05
NaOCl vs. CHX	3599	*p* > 0.05	22,727	*p* < 0.001
